# KRAS, BRAF genotyping reveals genetic heterogeneity of ovarian borderline tumors and associated implants

**DOI:** 10.1186/1471-2407-13-483

**Published:** 2013-10-18

**Authors:** Sabine Heublein, Katinka Grasse, Harald Hessel, Alexander Burges, Miriam Lenhard, Jutta Engel, Thomas Kirchner, Udo Jeschke, Doris Mayr

**Affiliations:** 1 Department of Gynecology and Obstetrics, Ludwig-Maximilians-University of Munich-Campus Innenstadt, Munich, Germany; 2 Department of Pathology, Ludwig-Maximilians-University of Munich, Munich, Germany; 3 Department of Gynecology and Obstetrics, Ludwig-Maximilians-University of Munich-Campus Grosshadern, Munich, Germany; 4 Department of Biostatistics and Epidemiology, Munich Tumor Registry, Ludwig-Maximilians-University of Munich, Munich, Germany

**Keywords:** *KRAS*, *BRAF*, Serous ovarian borderline tumor, Implants

## Abstract

**Background:**

Patients diagnosed for a serous ovarian borderline tumor (s-BOT) typically present with an excellent clinical outcome. However there have been controversies concerning the prognostic impact of so-called implants, an extra ovarian spread occurring alongside the s-BOT in certain cases. It remains obscure whether these implants actually resemble metastasis owning the same genetic pattern as the ovarian primary or whether they develop independently.

**Methods:**

The current study, in the aim of further clarifying the genetic origin of implants, assessed *BRAF/KRAS* hot spot mutations and the p53/p16^INK4a^ immunophenotype of s-BOTs and corresponding implants (n = 49) of 15 patients by pyro-sequencing and immunostaining, respectively.

**Results:**

A significant proportion of both s-BOTs and implants showed *KRAS* or *BRAF* mutation and though p16^INK4a^ was found to be abundantly expressed, p53 immunoreactivity was rather low. When genotypes of *BRAF/KRAS* mutated s-BOTs and corresponding implants were compared no patient presented with a fully matching mutation profile of s-BOTs and all corresponding implants.

**Conclusions:**

The current study reveals genetic heterogeneity of s-BOTs and implants, as none of the markers examined showed constant reciprocity. Hence, our findings may assist to explain the different clinical presentation of s-BOTs and implants and might encourage to applying more individualized follow up protocols.

## Background

Serous ovarian borderline tumors (s-BOTs) and advanced stage invasive ovarian cancer (IOC) differ regarding morphological, clinical and molecular characteristics. s-BOTs show an atypical degree of proliferation lacking obvious stromal invasion [1]. According to the Malpica grading system s-BOTs may be associated with low-grade IOC [2], while high grade IOCs show marked nuclear atypia and mitotic activity [2].

Usually s-BOTs are characterized by their excellent clinical outcome as compared to advanced stage IOC [3,4]. Though, it needs to be noted that, in contrast to IOC, s-BOTs frequently affect younger patients and might, in certain rare but not insignificant cases, also progress into low grade IOC [1,5]. Since it remains challenging to identify patients at risk, it has been discussed repeatedly, to which extent so called implants, representing extra-ovarian lesions coincidentally occurring in about 20% of particularly serous s-BOT cases, influence patients’ prognosis [1,4,6]. While it is broadly accepted that implants presenting with invasive features are of adverse prognostic significance [7-9], the impact of non-invasive implants is less clear. As stated by the WHO non-invasive implants have no to little effect on patients’ outcome, while invasive implants are associated with increased recurrence rates and a significantly reduced 10 year survival [10]. Hence it is critical to further investigate implant pathophysiology and genetic origin.

It remains to be elusive whether implants actually arise independently alongside the ovarian s-BOT as part of a field effect, or whether they may directly develop from the ovarian primary resembling its metastasis. Within the first scenario implants are supposed to be of heterogeneous origin and thus comprise a different genetic pattern as compared to the ovarian tumor while metastases are postulated to rise in a clonal manner and thus should closely mimic their primary. In general, since clonality of neoplastic lesions is discussed to be of prognostic significance, determining the mutation status of s-BOT and their corresponding implants may turn out to be of clinical use.

To address this question, this study employed pyrosequencing of *KRAS* (Kirsten rat sarcoma viral oncogene homolog) and *BRAF* (v-raf murine sarcoma viral oncogene homolog B1) hot spot regions in s-BOTs and corresponding implants. Since both *KRAS* and *BRAF* are known to be frequently mutated in s-BOTs [11], they are especially suitable to indicate a possible genetic descent of extraovarian implants in s-BOT patients. BRAF and KRAS are upstream activators of the mitogen-activated protein kinase (MAPK) cascade which is commonly hyper-activated in different types of human cancer [12].

Further, p16^INK4a^ (p16) and p53 immunoreactivity of s-BOTs and associated implants was compared. p16 acts as a cell cycle inhibitor antagonizing MAPK signaling and is compensatory up-regulated under hyper-proliferative conditions including high risk human papilloma virus infection or oncogene activation [13-15]. Accumulation of the tumor suppressor protein p53 was observed in malignant cells [16] thus leading to the assumption that mutation in *TP53* may cause overexpression of p53 protein [16,17]. Up to now the mechanism leading to p53 up-regulation remains to be controversial [17]. Today, assessing p53 by immunohistochemistry instead of *TP53* mutation analysis is a well-established method [18-21] and has been intensively studied [22,23]. However, it needs to be mentioned that so far p53 immunohistochemistry may not fully resemble *TP53* mutation testing. Though high grade IOC is characterized by p53 overexpression, the latter is considered a seldom event in both low grade IOC [24,25] and in s-BOTs [26]. We included both p16 and p53 immunohistochemistry in order to investigate whether these markers might be useful to match implants and their corresponding s-BOT(s).

Ultimately, our goal was to clarify whether implants actually resemble the mutation (regarding *KRAS* and *BRAF*) or protein expression (regarding p16 and p53) profile of corresponding s-BOTs. Further insights on origin and genetic causes of both s-BOTs and corresponding implants may help to identify patient subgroups that might benefit from more individualized therapy.

## Methods

### Patients

In total 15 patients (Table 1), that had undergone surgery at the Department of Obstetrics and Gynecology of the Ludwig-Maximilians-University of Munich due to a suspected ovarian tumor between 2003 and 2009, were included in this study. All patients were diagnosed for either uni (n = 9)- or bilateral (n = 6) s-BOTs and concomitant implants. One up to 19 implants (total number: n = 49) were identified in each patient. Patient age at surgery ranged between 22 and 75 years (median = 46 years). Histological diagnoses according to the FIGO criteria were conducted at the Department of Pathology of the Ludwig-Maximilians-University of Munich by two experienced gynecological pathologists. All tumors were of serous histology and were staged analogically to invasive carcinomas of the ovary. Five patients were classified as FIGO II, while the remaining 10 patients were staged as FIGO III.

**Table 1:** Genotypes of s-BOTs and corresponding implants

	**s-BOT(s)**			**Implant(s)**			
**Patient**		***KRAS***	***BRAF***	**Implants (n)**	**Mutation profiles (n)**	***KRAS***	***BRAF***
1	Unilateral	wt	wt	1	1	wt	wt
2	Bilateral	wt	p.V600E	4	3	wt	wt
					1	wt	p.V600E
		wt	p.V600E				
3	Unilateral	p.G12D	wt	1	1	p.G12D	p.V600E
4	Unilateral	wt	wt	4	4	wt	wt
5	Bilateral	wt	p.V600E	2	1	wt	wt
					1	p.G12D	wt
		wt	p.V600E				
6	Unilateral	wt	p.V600E	2	1	wt	wt
					1	wt	p.V600E
7	Unilateral	wt	wt	3	3	wt	wt
8	Bilateral	wt	wt	2	2	wt	wt
		wt	wt				
9	Unilateral	wt	p.V600E	2	1	wt	p.V600E
					1	wt	wt
10	Unilateral	p.G12A	p.V600E	1	1	wt	p.V600E
11	Bilateral	wt	p.V600E	2	1	p.G12V	p.V600E
					1	wt	p.V600E
		p.G12V	wt				
12	Bilateral	p.G12A	p.V600E	3	1	p.G12A	p.V600E
					2	wt	p.V600E
		p.G12A	p.V600E				
13	Unilateral	wt	wt	2	2	wt	p.V600E
14	Unilateral	p.G12V	wt	1	1	wt	p.V600E
15	Bilateral	p.G12V	p.V600E	19	11	p.G12V	wt
					1	wt	p.V600E
		p.G12V	wt		1	p.G12V	p.V600E
					6	wt	wt

n.a. = not applicable. The total number of implants diagnosed in each patient is displayed in the “number of implants” column and the count of how often each mutation profile was observed is specified in the “mutation profiles” column.

All implants were non-invasive according to the WHO criteria and presented with serous histology. In four cases the patient had one implant, in six cases two and in the remaining cases three or more. Differentiation between non-invasive and invasive implants was performed according to criteria of the WHO [10] by two experienced gynecological pathologists at the Department of Pathology of the Ludwig-Maximilians-University of Munich. According to the WHO, the diagnosis of non-invasive implants was performed when they were typically localized on the surface, in submesothelial spaces or with extension into interlobular fibrous septa without infiltration of the underlying tissue. In contrast, diagnosis of invasive implants was made when the lesions disorderly infiltrated the normal tissue with irregular borders and showed nuclei resembling cells of low-grade serous adenocarcinoma.

Follow up data of all patients were available and retrieved from the Munich tumor registry. As of September 2013 three patients from the cohort had already died at the age of 78 years, 75 years and 73 years. Since just one of these deaths was reported as being cancer related, the remaining two cases were excluded from survival analysis. Mean follow up was 4.8 years (95% CI = 3.5 years - 6.2 years) and the only cancer related death was observed in a woman that died at the age of 78 (2.6 years after surgery).

### Ethics statement

The study was approved by the ethics committee of the LMU of Munich. Patients’ data and samples were anonymized and processed in compliance with the guidelines of the Helsinki Declaration of 1975.

### Immunohistochemistry (IHC)

Sections of standard paraffin-embedded tissue were stained for p53 (ThermoScientific, Munich, Germany and p16 (CINtec®Histology, Roche, Mannheim, Germany using Ventana Benchmark® XT (Roche) in an automatic manner. The signal was quantified using a semi quantitative method [27] by two independent observers by consensus. At a glance the immuno-reactive (IR)-score quantifies intensity (1 = low, 2 = moderate, 3 = strong) and percentage of stained cells (0 = no, 1 = less than 10%, 2 = 10%-50%, 3 = 51%-80%, 4 = 81%-100%). Multiplication of these scores results in the IR-score ranging from 0 to 12. In this study the IR-score was subdivided as follows: IRS = 0, IRS = 1, IRS = 2 - negative; IRS = 3, IRS = 4 - weakly positive; IRS = 6, IRS = 8 - moderately positive; IRS = 9, IRS = 12 - strongly positive.

### *KRAS/BRAF* pyrosequencing

Hot spot mutations in KRAS exon 2 and BRAF exon 15 were analyzed. For each s-BOT/implant sequencing analysis of *KRAS* and *BRAF* was done on the same anatomically micro-dissected tumor/implant sample. *KRAS*/*BRAF* genotyping was performed by PCR and direct sequencing in a German reference laboratory for KRAS mutation testing (Department of Pathology, LMU of Munich). All tumors/implants underwent micro-dissection, followed by DNA isolation using DNA Micro-Amp-kits (Qiagen, Hilden, Germany) according to the manufacturer’s protocol. Mutation testing in codons 12 and 13 of the KRAS proto-oncogene was done by pyrosequencing employing Qiagen’s PyroMark GoldVR kits together with a Q24 pyrosequencer device (Qiagen). This procedure was used to detect mutations in the KRAS proto-oncogene with a specificity of 0.98 and sensitivity of 0.99 [28,29].

Following DNA isolation *BRAF* exon 15 was amplified [PCR buffer, 1.5 mM MgCl_2_, 200 nM dNTPs, 400 nM primers, 1 U Hotstar Taq-polymerase (Qiagen)] using the following primers: forward 5’-TGAAGACCTCACAGTAAAAATAGG-3’, reverse 5’- TCCAGACAACTGTTCAAACTGAT-3’. PCR products were processed using Pyro-Gold kits (Qiagen) together with 3 nM of the corresponding sequencing primer employing the PyroMark Q24 device (Qiagen). The PyroMark™-Q24 software (Qiagen) was used for data analysis.

### Statistical analysis

For all statistical calculations Superior Performance Software System 19 was used. Wilcoxon Signed Ranks Test, Mann–Whitney U Test and the Spearman correlation coefficient were employed to analyze data. Values are displayed in terms of mean ± standard error and p-values lower than 0.05 were considered as statistically significant.

## Results

### p53 and p16 in s-BOTs and implants

**Figure 1:**
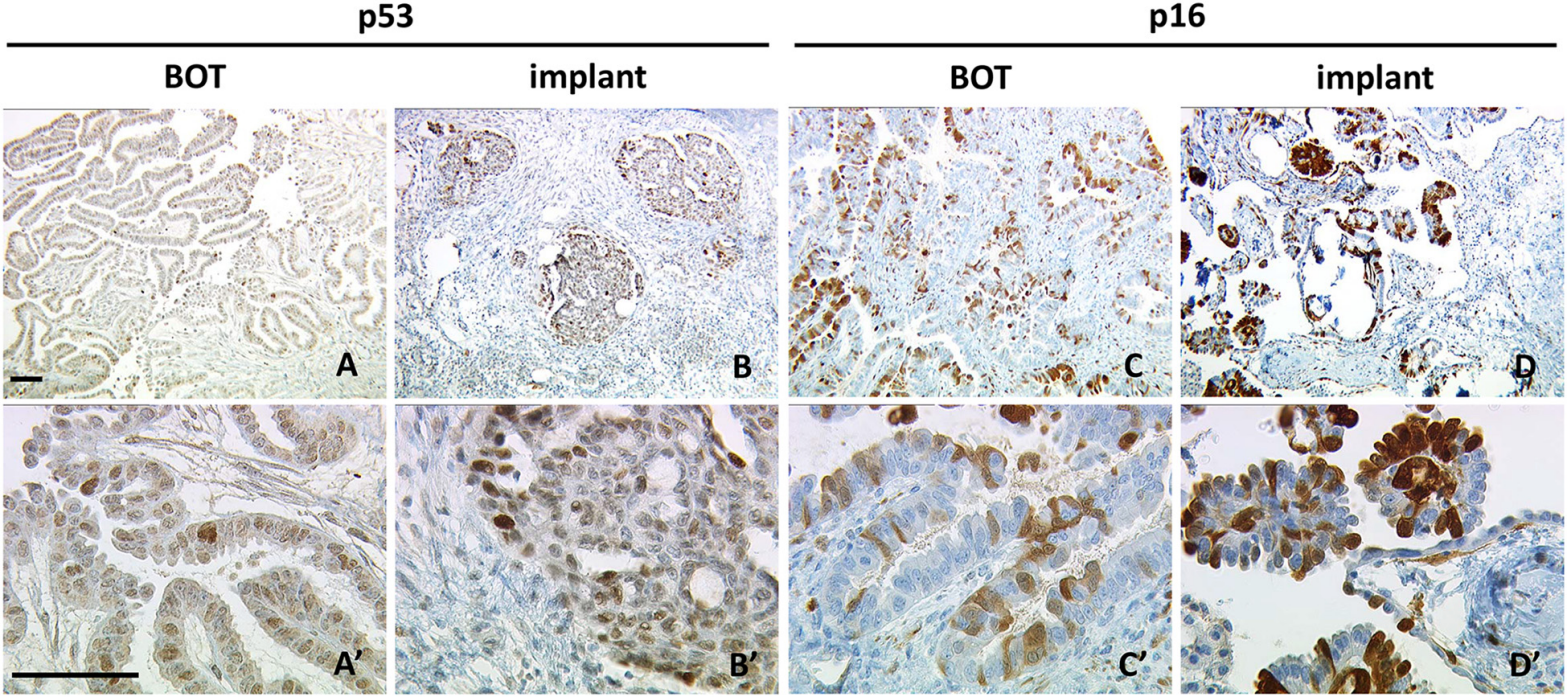
**Representative microphotographs of immuno-histochemical p53 (A, A’, B, B’) and p16 (C, C’, D, D’) staining are shown. A**, **A’**, **C**, **C’**: serous ovarian borderline tumor (s-BOT); **B**, **B’**, **D**, **D’**: implants associated with s-BOT(s). Scale bars in **A**/**A’** equal 100 μm and apply to **A**-**D**/**A’**-**D’**.

None of the s-BOT samples examined was rated as highly (IRS > 8) positive for p53. Less than half of all patients were found to carry at least one s-BOT rated as either weakly (n = 5; 5/15; 33.3%) or moderately (n = 2; 2/15; 13.3%) positive for p53 and in eight (8/15; 53.3%) cases p53 was not detected at all. In contrast, p16 was abundantly expressed with the majority of patients showing up to strong (n = 4; 4/15; 26.7%), up to moderate (n = 5; 5/15; 33.3%) or at least weak (n = 4; 4/15; 26.7%) p16 positivity. Consequently, the overall immunoreactivity level for p16 was significantly higher (mean IRS = 6.0 ± 0.8 vs. mean IRS = 2.5 ± 0.4; p = 0.001) than for p53. Immunoreactivity of p53 and p16 (Figure 1) did not correlate and none of the both was significantly associated with clinical tumor staging.

Immunohistochemical analysis of p53 in implants (Figure 1) revealed strong p53 positivity in one (1/49; 2.0%), moderate in another one (1/49; 2.0%) and weak in nine (9/49; 18.4%) implant samples. In terms of patients, only one patient was identified with an implant strongly expressing p53. This patient (#3 in Table 1, Additional file 1) presented with an implant also strongly expressing p16. Further this implant was found to carry both *KRAS* p.G12D and *BRAF* p.V600E at the same time. Moreover another seven patients showed either up to moderate (one patient with moderate p53 expression in one implant) or up to weak (six patients with at least one weakly stained implant in each patient) positivity for p53, respectively. No p53 positive implant at all could be identified in the remaining seven cases. Yet again overall immunoreactivity for p53 was significantly lower than for p16 (mean IRS = 1.7 ± 0.3 vs. mean IRS = 5.4 ± 0.6; p < 0.001), though regarding implants expression of the two correlated (p = 0.032). About one third (15/49; 30.6%) of implants was found to be negative for p16. Twelve implants were weakly positive (12/49; 24.5%) for p16, while 22 (22/49; 44.9%) implant samples were identified as highly or moderately expressing p16, respectively. In respect to patients, nine (9/15; 60.0%) of them were diagnosed with at least one implant overexpressing p16.

### KRAS/BRAF genotypes in s-BOTs and implants

**Figure 2:**
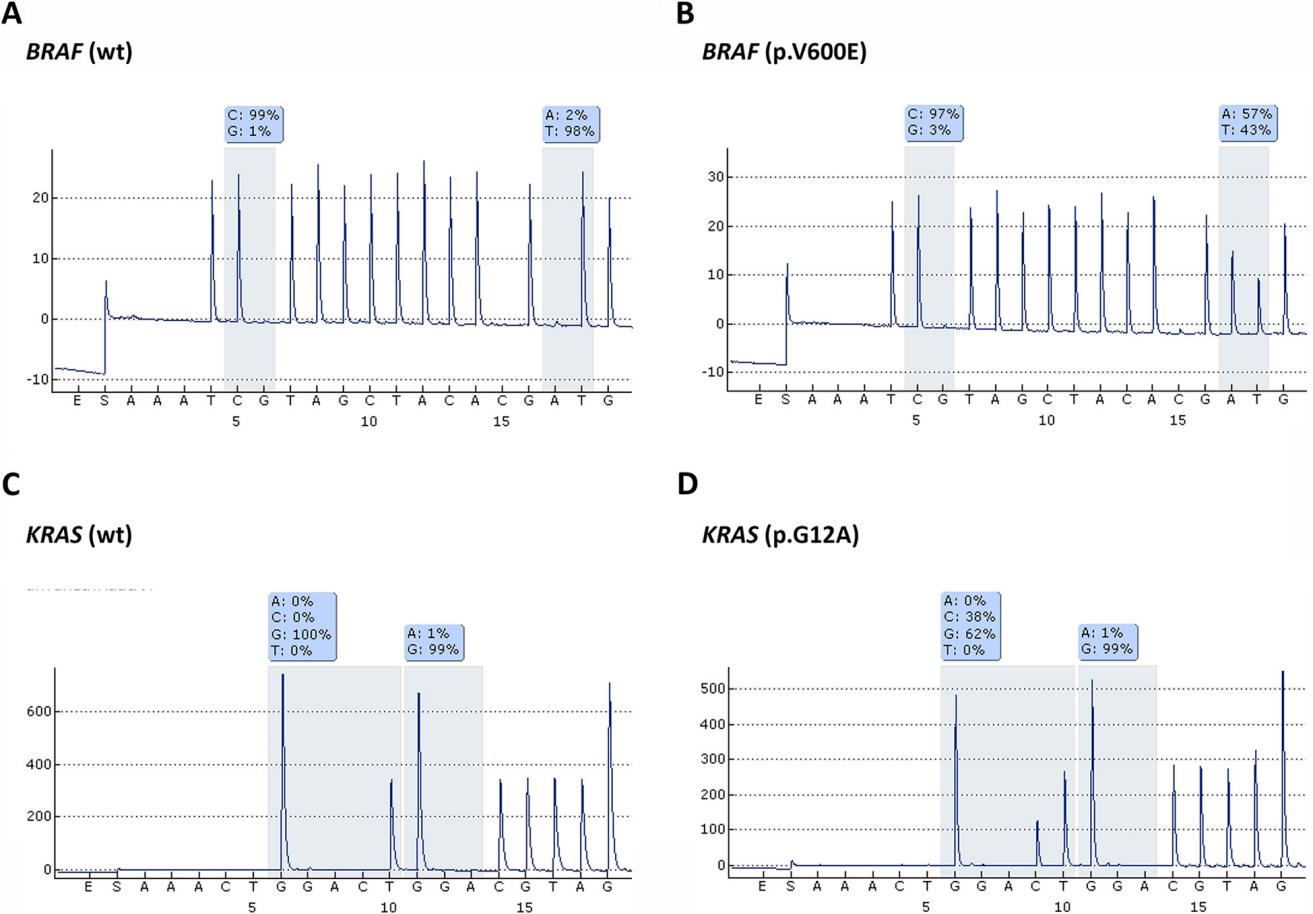
**Representative pyro-sequencing mutation analysis results are shown.** The *BRAF* wildtype allele **(A)** is frequently mutated (**B**; p.V600E) in s-BOTs and implants. An amino acid substitution at codon 12 in *KRAS* (**D**; p.G12A) alters wildtype *KRAS***(C)**.

KRAS/BRAF genotypes were determined by pyrosequencing in s-BOTs and implants (Figure 2). Regarding the ovarian primary the *BRAF* variant p.V600E was observed in at least one ovary of about half of all patients (8/15; 53.3%) while *KRAS* alterations (p.G12V, p.G12D, p.G12A) were detected in six patients (6/15; 40.0%; Table 1). Just one patient with a bilateral s-BOT did not show either *KRAS* or *BRAF* mutation. A combined *KRAS*-*BRAF* mutation in the same s-BOT was detected in three patients (3/15; 20.0%) while another patient was identified with single *KRAS* p.G12V in the s-BOT of the left ovary and single *BRAF* p.V600E in the s-BOT of the right ovary. *BRAF* or *KRAS* mutated tumors were not significantly different in respect to their p53, p16 immunophenotype. Moreover, no relation of *KRAS* or *BRAF* mutation and clinical tumor stage was observed.

When implants were analyzed, about one third (16/49, 32.7%) of all implant samples presented a single point mutation in codon 12 of the *KRAS* gene (p.G12V: 13/49, 26.5%, p.G12D: 2/49, 4.1%, p.G12A: 1/49, 2.0%). The *BRAF* sequence variation p.V600E was detected in 15 (15/49, 30.6%) implant samples. Regarding total implant count (n = 49) a co-existing *KRAS* and *BRAF* mutation per sample was detected in 4 (4/49, 8.2%) implants. BRAF mutated implants showed a trend (p = 0,057) of higher overall p16 immunoreactivity though no such relation was observed for p53.

Patient wise five patients (5/15, 33.3%) were found to carry a *KRAS* mutation in at least one implant while *BRAF* p.V600E was detected in ten (10/15, 66.7%) patients. A coexisting mutation of *KRAS* and *BRAF* was observed in implants of four (4/15, 26.7%) patients and four (4/15, 26.7%) presented only without either *KRAS* or *BRAF* aberrations in their implants regarding the gene loci studied.

### Comparison of s-BOTs and corresponding implants

To address the question whether implants are developing alongside the ovarian primary or whether they directly spread from there, s-BOTs and their corresponding implants were compared regarding p53, p16 expression and *KRAS*, *BRAF* genotype. By contrasting s-BOT cases and their implants we found a strong correlation in terms of mean p16 (p16 [s-BOT] - p16 [implant]: p = 0.006; Table 2) but not p53 mean immunoreactivity.

**Table 2:** Spearman-correlation of mean p53 and mean p16 expression in s-BOTs and implants

		**p53-s-BOT (mean IRS)**	**p16-s-BOT (mean IRS)**
**p16-implant (mean IRS)**	cc	.026	.673^**^
	p (2-tailed)	ns	**.006**

In case of more than one implant or in case of bilateral s-BOT mean IR-scores were used to correlate s-BOTs and associated implants. ns = not significant, cc = correlation coefficient (Spearman’s rho), ** Correlation is significant at the 0.01 level (2-tailed) and significant results are shown in bold.

Out of the 15 patients examined within this study four cases were found to show wildtype genotypes regarding both *BRAF* and *KRAS* in their s-BOTs as well as in all the implants diagnosed in these particular patients.

Four out of the six cases that had been diagnosed for a *KRAS* mutation of their s-BOTs presented with a matching *KRAS* mutation in at least one implant while a *KRAS* mutation different from the one found in the s-BOT was not detected. A complete match of a mutant *KRAS* allele in s-BOTs and all implants was just observed in a single patient that notably had only one implant at all and did not match regarding the *BRAF* allele. One patient in this study carried the *KRAS* p.G12D allele in an implant though no *KRAS* mutation at all was detected in the corresponding s-BOTs. Vice versa, two patients presented with a *KRAS* mutated s-BOT though their implants only carried the *KRAS* wildtype allele.

In seven out of eight patients diagnosed with a *BRAF* mutated s-BOT the same *BRAF* mutation was found in at least one implant. Notably, four of these patients (*BRAF* mutated s-BOT and the same *BRAF* mutation in at least one implant) also carried implants that were found to have a *BRAF* wildtype genotype. In one case *BRAF* p.V600E was not detected in any implant, though *BRAF* p.V600E was found in the s-BOT of this patient. The other way round three patients only carried *BRAF* mutated implant(s) though the ovarian lesion was homozygous for the wildtype allele.

In conclusion, when genotypes of *BRAF/KRAS* mutated s-BOTs and corresponding implants were compared no patient presented with a fully matching *BRAF/KRAS* mutation profile of s-BOTs and all implants observed in the particular case (Table 1).

## Discussion

### p53/p16 and its relation to KRAS/BRAF genotype

Advanced stage IOCs are supposed to initiate from *TP53* mutated ovarian surface [30] or fallopian tube [31] epithelium. As mutation in *TP53* may cause its up-regulation, protein over-expression of p53 is frequently assessed [18-21]. This study performed immunohistochemistry to determine p53 up-regulation and defined p53 overexpression for strongly positive (IRS > 8) cases. Unlike p53, the cell cycle inhibitor p16 is routinely assessed to sub-classify certain neoplastic lesions. Physiologically, p16 acts as tumor suppressor inhibiting cell cycle progression hence attenuating mitogenic effects. Cellular stress factors like for instance oncogenic activation, as mediated by HPV infection or constitutive activation of mitogenic pathways (e.g. *KRAS* mutation) trigger compensatory p16 up-regulation [13-15]. This study detected a trend of higher p16 expression in *BRAF* mutated implants leading to the conclusion that p16 may act to attenuate *BRAF* induced cell cycle progression signals. When s-BOTs and their corresponding implants were contrasted regarding *KRAS* and *BRAF* mutation status. s-BOTs and implants correlated in respect of p16 expression. A significant proportion of s-BOTs and implants investigated here were negative for *KRAS* and/or *BRAF* mutation anticipating that in patients without *KRAS* or *BRAF* mutations other genetic events are likely to contribute to s-BOT development and implant formation. Regardless the fact that aberrations in *KRAS* and *BRAF* had been closely associated with development and progression of s-BOTs [32-37], other oncogenic routes, e.g. mutation of p53, being capable to initiate malignant transformation, need to be speculated for s-BOTs carrying *KRAS/BRAF* wildtype alleles. Yet, regarding s-BOTs in this study neither expression of p53 nor of p16 was significantly altered comparing *KRAS/BRAF* mutated vs. wildtype s-BOTs. These findings lead to the conclusion that even in absence of mutated *KRAS*/*BRAF*, initiation of s-BOTs is not reliant on p53 or may necessarily alter p16 expression.

### Genetic heterogeneity of s-BOTs and associated implants

In contrast to *BRAF/KRAS*, mutations in *TP53* are reported to be rare in s-BOTs. Comparable to others [26], this study did not detected strong immunoreactivity for p53 in any s-BOT case, confirming thus the hypothesis that s-BOTs and advanced stage IOCs arise via different genetic pathways. Unexpectedly, herein coexisting *BRAF* and *KRAS* mutations were observed. This finding is unlikely to be due to sequencing inconsistencies, as the methods employed to determine BRAF and KRAS mutation status had been intensively validated [28,38,39]. KRAS mutation analysis was taken out at a German reference laboratory for KRAS mutation testing at our institute. Though coexistence of mutations occurring in *BRAF* or *KRAS* has been assumed to be mutually elusive, such phenomena were recently observed in colorectal adenoma/cancer [40,41] and ovarian malignancies [42,43]. Implant formation is a relatively seldom event in s-BOT genesis. However, since just s-BOT patients diagnosed with concomitant implants were included in the current study, it is hard to compare our data to studies mostly reporting on BOTs in general (regardless of the diagnosis of implants). A constitutive activation of two directly coupled downstream signaling partners in the same pathway is unusual. This is why we assume that coexisting *KRAS*, *BRAF* mutations in the same s-BOT may be indicative for a secondary genetic event or may reflect a possible polyclonal origin of s-BOTs and implants.

Extraovarian lesions associated with s-BOTs are referred to as implants, which present as small nodules mostly located on the omentum and peritoneal surfaces. For other neoplasias such a spread beyond the tumor is termed metastasis, assuming that cells initiating it have originally settled there from the primary tumor. Indeed, it is widely unknown whether implants actually rise as metastasis of the primary ovarian neoplasm or whether they rather represent *in situ* lesions of extraovarian tissue. The latter hypothesis would presume different, distinct genetic changes characterizing implants vs. s-BOTs, indicating that they have developed independently. The current study addressed this question by comparing s-BOTs and corresponding implants regarding genetic alterations associated with initiation of ovarian tumors. Since full penetrance of either *KRAS* or *BRAF* aberrations was not observed in any patient, our data suggest that s-BOTs and implants develop independently and possibly do not derive from the same precursor lesion. Most studies undertaken so far used hyper-methylation analysis to determine tumor clonality and agree on the finding that s-BOT and corresponding implants show mono- as well as polyclonal descent [44,45]. In contrast to IOC [46-48] it has been suggested earlier that s-BOT are of multifocal genesis and that associated extraovarian tumors rise independently [44,45,49]. Accordingly, the present study strongly supports multifocal origin of s-BOTs and their associated implants as no fully matching mutation profile among s-BOTs and their corresponding implants were observed. In order to prove this, we employed state of the art mutation analysis and immune profiling. Taking into consideration that clonal descent would imply the presence of a common genetic pattern, our data prove that at least some implants may have risen independently from the ovarian malignancy diagnosed in the same patient. Statistical association of p16 immunoreactivity in implants and the corresponding s-BOT(s) may reflect the fact that p16 is regulated by external triggers like for instance virus mediated oncogenic activation or stimulation of mitogenic pathways [13-15]. These may similarly affect both s-BOTs and implants hence provoking similar secondary events (e.g. compensatory p16 up-regulation) that not necessarily claim to be linked to s-BOT/implant origin. Since studies on the genetic descent of implants only employed small patient numbers, it is imperative to evaluate this topic on a larger scale in order to validate our conclusions.

Malignant transformation of non-invasive implants and hence worsening of clinical presentation is a process depending on time and requires a minimum 10 year follow up [3,4] period. Due to the fact that the follow up of the cohort studied herein is relatively short, statistical survival analysis has not been performed. Nevertheless our finding that s-BOTs and associated implants are heterogeneous lesions may explain a different clinical presentation of s-BOTs and implants and might encourage to applying more individualized follow up protocols.

## Conclusions

By contrasting *BRAF/KRAS* genotypes and p53/p16 expression profiles of s-BOTs and their corresponding implants this study revealed genetic heterogeneity of the two. When genotypes of *BRAF/KRAS* mutated s-BOTs and corresponding implants were compared, no patient presented with a fully matching mutation profile of s-BOT and all corresponding implants, hence hypothesizing that s-BOTs and implants are not likely to arise from a common precursor lesion.

## Competing interests

The authors declare no conflict of interests.

## Authors’ contributions

SH significantly contributed to data interpretation, statistical analysis and drafted the manuscript. KG and HH performed the experiments and significantly contributed to data analysis. Design and coordination of the study was done by DM, AB, ML, JE and UJ. DM initiated and supervised the study. All authors read and approved the final version of the manuscript.

## Pre-publication history

The pre-publication history for this paper can be accessed here:

http://www.biomedcentral.com/1471-2407/13/483/prepub

## Supplementary Material

Additional file 1: Figure S1 A microphotograph of strong immuno-histochemical p53 staining is shown p53 was found to be strongly expressed in an implant detected in patient #3. Scale bar equals 100 μm. Click here for file
