# Supplementary figures and images for: KRAS, BRAF genotyping reveals genetic heterogeneity of ovarian borderline tumors and associated implants
Source: BMC Cancer. 2013 Oct 18;13:483. doi: 10.1186/1471-2407-13-483 (PMC4015926; doi:10.1186/1471-2407-13-483)

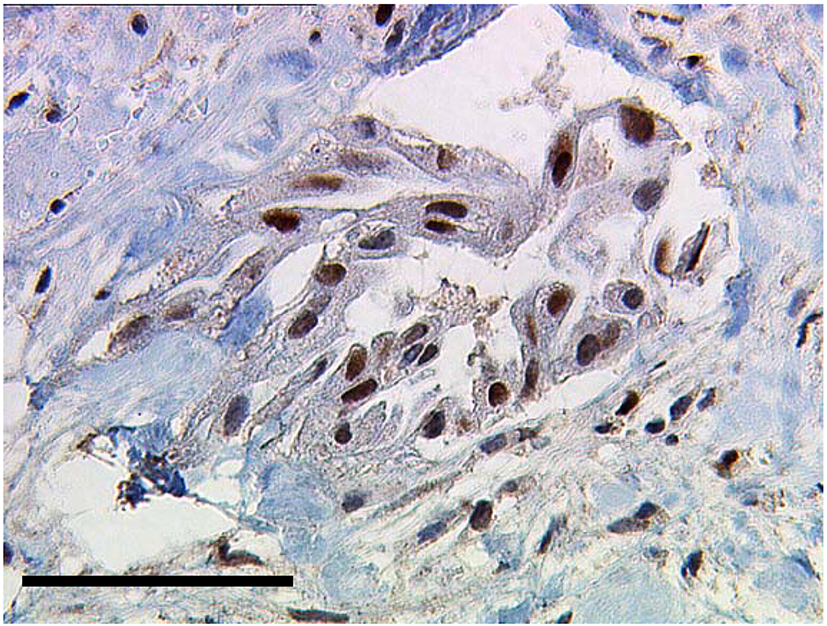

Supplement: Additional file 1: Figure S1 — A microphotograph of strong immuno-histochemical p53 staining is shown p53 was found to be strongly expressed in an implant detected in patient #3. Scale bar equals 100 μm. [file 1471-2407-13-483-S1.tiff]
